# Effects of dietary oil sources and fat extraction methods on apparent and standardized ileal digestibility of fat and fatty acids in growing pigs

**DOI:** 10.1186/s40104-022-00798-w

**Published:** 2022-12-14

**Authors:** Lu Wang, Wenjun Gao, Junyan Zhou, Huangwei Shi, Tenghao Wang, Changhua Lai

**Affiliations:** 1 State Key Laboratory of Animal Nutrition, Ministry of Agriculture Feed Industry Centre, China Agricultural University, Haidian District, No. 2 Yuanmingyuan West Road, Beijing, 100193 P. R. China; 2 Beijing Bio-Feed Additives Key Laboratory, China Agricultural University, Beijing, 100193 P. R. China; 3 Zhejiang Qinglian Food Co., Ltd., Jiaxing, 314399 P. R. China

**Keywords:** Fat extraction methods, Fatty acids, Growing pigs, Ileal digestibility, Oil sources

## Abstract

**Background:**

There is a lack of data for the standardized ileal digestibility (SID) of fat and fatty acids in national feed databases. In addition, it is important to specify the procedures used for fat analyses. Therefore, an experiment was conducted to 1) determine the apparent ileal digestibility (AID) and SID of fat and fatty acids in ten different oil sources for growing pigs and to develop prediction equations for SID of fat based on fatty acid composition; and 2) compare the effect of the fat extraction methods on the calculated values for endogenous loss and digestibility of fat.

**Methods:**

Twenty-two barrows (initial body weight: 32.1 ± 2.3 kg) were surgically fitted with a T-cannula in the distal ileum, and allotted to 1 of 11 experimental diets in a 4-period Youden Square design. A fat-free diet was formulated using cornstarch, soy protein isolate and sucrose. Ten oil-added diets were formulated by adding 6% of dietary oil sources to the fat-free diet at the expense of cornstarch. All diets contained 26% sugar beet pulp and 0.40% chromic oxide.

**Results:**

The endogenous loss of ether extract (EE) was lower than that of acid-hydrolyzed fat (AEE; *P* < 0.01). There were significant differences in the AID and SID of fat and saturated fatty acids across the dietary oil sources (*P* < 0.05). The SID of AEE for palm oil was lower than that of sunflower oil, corn oil, canola oil, rice oil and flaxseed oil (*P* < 0.01). The AID and SID of fat ranged from 79.65% to 86.97% and from 91.14% to 99.18%. Although the AID of EE was greater than that of AEE (*P* < 0.01), there was no significant difference in SID of EE and AEE except for palm oil. The ratio of unsaturated to saturated fatty acids (U/S) had a positive correlation with SID of fat (*P* < 0.05), whereas C16:0 and long chain saturated fatty acids (LSFA) were significant negatively correlated with SID of fat (*P* < 0.01). The best-fit equation to predict SID of fat was SID AEE = 102.75 − 0.15 × LSFA − 0.74 × C18:0 − 0.03 × C18:1 (Adjusted coefficient of determination = 0.88, *P* < 0.01).

**Conclusions:**

When calculating the SID of fat, the EE content of the samples can be analyzed using the direct extraction method, whereas the acid hydrolysis procedure should be used to determine the AID of fat. Fat digestibility of dietary oils was affected by their fatty acid composition, especially by the contents of C16:0, LSFA and U/S.

**Supplementary Information:**

The online version contains supplementary material available at 10.1186/s40104-022-00798-w.

## Introduction

Supplementing oils in pig diets not only increases the energy density of the diet and provides essential fatty acids, but also promotes the absorption of fat-soluble vitamins, reduces dust and improves the palatability of the diet [1–3]. Considering the high prices of dietary oils, the nutritive value of different oil sources should be accurately evaluated to achieve precise nutrition. It is well known that the digestibility of nutrients, in practical feed-ingredient assessment, is usually used to determine the proportion of nutrients that is absorbed [4]. The standardized ileal digestibility (SID) of fat and fatty acids is more reflective of availability of dietary oils in pigs. The terminology and determination method of SID of fat and fatty acids need to be further clarified.

The oil sources used in animal diets are diverse in fatty acid composition, which may influence the digestion, absorption and metabolic utilization of dietary oils [5]. Saturation degree, carbon chain length and positional distribution of fatty acids, and free fatty acid content greatly influence the potential for micelle formation by affecting the concentration of mixed bile-salt lipid micelles in the intestinal lumen [6, 7]. Studies correlating the effect of individual fatty acids on dietary oil digestibility are very limited.

Previous studies have successfully established prediction equations for energy values of dietary oil sources in pigs [8–10]. The development of such prediction equations potentially reduces the need for expensive and time-consuming animal trials. Unfortunately, there is no available information on the prediction of SID of fat in oils for growing pigs. In addition, the procedure used to analyze fat (direct extraction or acid hydrolysis) may influence the calculated values for endogenous loss and digestibility of fat. However, no studies have comprehensively compared the effect of extraction methods on endogenous loss and digestibility of fat. Selection of the appropriate laboratory method is critical for accurate assessment of fat digestibility.

Our previous study showed that correcting apparent ileal digestibility (AID) for basal endogenous losses of fat (ELF) and fatty acids (ELFA) obtained by the fat-free diet method can accurately estimate SID values [11]. Therefore, the objective of the current study was to determine the AID and SID of fat and fatty acids in different oil sources for growing pigs and to develop prediction equations for SID of fat based on fatty acid composition. These values are critical for the determination of fatty acid requirements. An additional objective was to compare the effect of the extraction methods of fat on the calculated values for endogenous loss and digestibility of fat.

### Materials and methods

All protocols used in the present experiment were reviewed and approved by the Institutional Animal Care and Use Committee of China Agricultural University (Beijing, China).

### Animals and experimental design

This experiment was conducted in the Metabolism Laboratory of Fengning Swine Research Unit at China Agricultural University (Academician Workstation in Chengdejiuyun Agricultural and Livestock Co., Ltd). Twenty-two crossbred barrows (Duroc × Landrace × Yorkshire; initial body weight: 32.1 ± 2.3 kg) were used in the experiment for 4 periods. Pigs were housed in individual stainless-steel metabolism crates (1.4 m × 0.7 m × 0.6 m) equipped with a stainless-steel feeder and a water nipple. Room temperature was maintained at 22 ± 2 ℃. Barrows were surgically fitted with a T-cannula in the distal ileum [12] and were allotted to 1 of 11 experimental diets in a 4-period Youden Square design which resulted in 8 observations per dietary treatment. Each experimental period lasted 10 d. Pigs had freedom of movement within the confines of their crates and free access to water throughout the experiment.

### Diets and feeding

The fat sources used in the present experiment contained sunflower oil, peanut oil, corn oil, canola oil, rice oil, soybean oil, palm oil, cottonseed oil, flaxseed oil and coconut oil, all of which were food grade (free fatty acids < 0.2%). Fatty acid composition of the ten oil sources were shown in Table 1. A fat-free diet was formulated from cornstarch, soy protein isolate and sucrose (Additional file 1), in which the fat content was negligible (Table 2; 0.29% acid-hydrolyzed fat; AEE, dry matter; DM basis). Ten oil-added diets were formulated by adding 6% of dietary fat sources to the fat-free diet at the expense of cornstarch. The same batch of sugar beet pulp (26%) was used for all experimental diets as a fiber source. The same proportion of vitamins, minerals and crystalline amino acids were supplemented across diets to meet or exceed nutrient recommendations of growing pigs as recommended by NRC [13]. Chromic oxide (0.40%) was contained in all diets as an inert marker.

**Table 1:** Fatty acid composition of dietary oil sources, % of total fatty acids

Fatty acid	Sunflower seed oil	Peanut oil	Corn oil	Canola oil	Rice oil	Soybean oil	Palm oil	Cottonseed oil	Flaxseed oil	Coconut oil
C6:0	−	−	−	−	−	−	−	−	0.00	0.45
C8:0	−	−	−	−	−	−	0.01	−	0.01	6.25
C10:0	−	−	−	0.01	0.00	−	0.02	−	−	5.38
C12:0	−	0.01	0.01	0.02	0.01	0.01	0.19	0.01	0.01	45.55
C14:0	0.07	0.04	0.03	0.06	0.24	0.08	0.97	0.74	0.05	19.29
C15:0	0.02	0.01	0.01	0.02	0.03	0.02	0.04	0.02	0.04	0.01
C16:0	7.03	8.76	12.01	4.21	18.16	11.02	39.76	22.33	5.66	10.23
C16:1	0.10	0.08	0.09	0.21	0.14	0.09	0.17	0.53	0.14	0.02
C17:0	0.04	0.08	0.07	0.04	0.05	0.10	0.10	0.09	0.12	0.01
C18:0	3.73	3.03	1.81	1.99	1.52	4.45	4.44	2.10	3.12	2.99
C18:1n9c	26.35	60.41	26.94	47.87	39.49	22.63	42.27	16.23	26.51	7.49
C18:2n6c	60.75	20.18	57.22	17.28	37.00	53.35	11.11	56.98	14.09	2.06
C18:3n3	0.28	0.09	0.60	7.44	1.27	6.82	0.16	0.21	48.87	0.02
C20:0	0.31	1.29	0.48	0.74	0.68	0.42	0.40	0.26	0.18	0.11
C20:1	0.18	1.53	0.29	6.52	0.64	0.23	0.16	0.09	0.46	0.06
C21:0	0.04	0.02	0.06	0.25	0.06	0.07	0.01	0.09	0.07	−
C20:4n6	−	−	−	−	−	−	−	−	0.11	−
C22:0	0.77	2.65	0.15	0.36	0.22	0.46	0.08	0.16	0.35	0.02
C22:1n9	0.01	0.17	0.02	12.30	0.07	0.02	−	0.01	0.04	−
C23:0	0.05	0.05	0.03	0.04	0.04	0.07	0.02	0.05	0.05	0.00
C24:0	0.26	1.59	0.15	0.18	0.35	0.17	0.09	0.12	0.10	0.05
C24:1	0.01	0.02	0.02	0.44	0.03	0.01	0.00	0.00	0.03	−
MCFA	−	0.01	0.01	0.03	0.01	0.01	0.22	0.02	0.03	57.63
LSFA	11.24	13.23	14.48	7.32	20.75	16.17	45.72	25.62	9.23	32.65
LUFA	87.67	82.46	85.17	91.63	78.60	83.13	53.87	74.04	90.22	9.65
SFA	12.32	17.52	14.81	7.93	21.37	16.87	46.13	25.96	9.75	90.35
MUFA	26.65	62.21	27.37	67.35	40.37	22.97	42.60	16.86	27.18	7.57
PUFA	61.03	20.26	57.82	24.72	38.26	60.17	11.27	57.19	63.07	2.08
UFA	87.68	82.48	85.19	92.07	78.63	83.14	53.87	74.04	90.25	9.65
U/S	7.11	4.71	5.75	11.62	3.68	4.93	1.17	2.85	9.25	0.11

*MCFA* Medium-chain fatty acids (6−12 carbons), *LSFA* Long-chain saturated fatty acids (13−21 carbons), *LUFA* Long-chain unsaturated fatty acids (13−21 carbons), *SFA* Saturated fatty acids, *MUFA* Monounsaturated fatty acids, *PUFA* Polyunsaturated fatty acids, *UFA* Unsaturated fatty acids, *U/S* The ratio of unsaturated to saturated fatty acids

− Below the limit of quantification

**Table 2:** Chemical analysis of experimental diets, % (dry matter basis)

Item	Fat-free diet	Oil-added diets									
		Sunflower seed oil	Peanut oil	Corn oil	Canola oil	Rice oil	Soybean oil	Palm oil	Cottonseed oil	Flaxseed oil	Coconut oil
Analyzed composition											
Dry matter	88.81	89.62	90.03	89.98	89.71	88.43	89.71	89.96	89.47	89.87	89.37
Ether extract	0.30	6.71	6.53	6.19	6.35	6.40	6.22	6.45	6.49	6.09	5.76
Acid-hydrolyzed ether extract	0.29	7.02	6.86	6.53	6.74	6.74	6.56	6.78	6.83	6.42	6.09
Crude protein	19.07	18.38	18.85	18.52	18.48	18.80	18.96	18.47	18.28	18.20	18.97
Neutral detergent fiber	11.31	10.66	10.29	10.43	10.86	10.72	10.65	11.87	11.71	11.19	11.14
Acid detergent fiber	5.88	5.45	5.08	5.21	5.45	5.48	5.45	5.75	5.87	5.48	5.60
Calculated composition											
ME, MJ/kg^a^	14.04	15.34	15.11	15.23	15.20	15.20	15.26	15.07	15.20	15.35	15.11

^a^ *ME* Metabolizable energy; Values were calculated according to the Chinese Feed Database (http://www.chinafeeddata.org.cn/admin/Zylist/index?type=cfdb2&year=2018)

Daily feed allowance for each collection period was adjusted. Barrows were weighed at the beginning of each period and supplied with experimental diets at 4% of their body weight. Diets were offered in two equal meals at 0800 and 1700 h daily. Water was available continuously for each pig. All diets were stored at 4 ℃ to prevent oxidative rancidity and were allowed to adjust to room temperature at least 12 h before being fed.

### Sample collection

Each experimental period involved 6 d for diet adaptation followed by 2 d for collection of feces and 2 d for collection of ileal digesta. On d 7 and 8, fresh fecal samples were collected into plastic bags as soon as possible after they appeared in metabolism crates throughout the day and were stored at −20 °C immediately [14]. On d 9 and 10, ileal digesta collection lasted for 9 h daily beginning at 0800 h [12]. During this period, ileal digesta samples were collected into plastic bags attached to the open cannula by cable ties. Bags were removed whenever they were half full of the digesta or at least every 30 min and then stored at –20 °C immediately. At the end of the experiment, fecal and ileal samples were thawed and pooled for each pig within experimental period, and a subsample was collected. Fecal and digesta subsamples were lyophilized in a vacuum-freeze dryer (Tofflon Freeze Drying Systems, Minhang District, Shanghai, China) and ground through a 1-mm screen for further chemical analysis.

### Chemical analyses

Experimental diets were analyzed for DM (method 930.15) [15], crude protein (CP) (method 990.03) [15], ether extract (EE) [16], AEE (method 954.02) [15], neutral detergent fiber (NDF) and acid detergent fiber (ADF), fatty acid profiles and chromium. The ADF and NDF were determined using F57 filter bags and fiber analyzer equipment (Fiber Analyzer; Ankom Technology, Macedon, NY, USA) according to the procedure of van Soest et al. [17] with the slight modification. The NDF was analyzed using heat stable α-amylase and sodium sulfite without correction for insoluble ash. The fatty acid profiles were determined using Gas Chromatography (6890 Series, Agilent Technologies, Wilmington, DE, USA) following a modification of the procedures of Sukhija and Palmquist [18]. The chromium concentration was determined using a polarized Zeeman Atomic Absorption Spectrometer (Hitachi Z2000, Tokyo, Japan) after nitric acid-perchloric acid wet ash sample preparation. Fecal and ileal digesta samples were analyzed for DM, EE, AEE, fatty acid profiles and chromium. Oil sources were analyzed for fatty acid profiles. All analyses were conducted in duplicate.

### Calculation

Added oil was the sole source of fat and fatty acids in the oil-added diets. Therefore, the AID and SID for fat and fatty acids in the test diet were equal to that of the oil. Similar to studies on amino acid digestibility of feed ingredients, the values of the endogenous losses, AID and SID of fat and fatty acids in the diet were calculated according to equations described by Stein et al. [19]. Furthermore, in the present experiment, the fat content of the sample was analyzed using two methods, resulting in two representations of the fat content, ether extract and acid hydrolyzed fat, both of which represent “fat” content.

The AID for fatty acid in each oil-added diet was calculated using the following equation:

$$AID=\left[1-\left(FAd/FAf\right)\times \left(Crf/Crd\right)\right]\times 100\mathrm{\%},$$

in which FAd and Crd were the concentrations of fatty acid and chromium in the ileal digesta (g/kg of DM) and FAf and Crf were the concentrations of fatty acid and chromium in the oil-added diet (g/kg of DM). The AID of EE and AID of AEE were calculated using the equation shown above.

The basal ELF and ELFA were measured from pigs fed the fat-free diet based on the following equation:

$$IFAend=\left[FAd\times \left(Crf/Crd\right)\right],$$

in which IFAend was the basal ELFA (g/kg DMI) and FAd and Crd were the concentrations of FA and Cr in the ileal digesta in the fat-free diet. The Crf was the concentration of chromium in the fat-free diet. The endogenous loss of ether extract and acid-hydrolyzed fat were determined using the equation shown above.

The SID for fatty acids in each oil-added diet was calculated by adjusting the AID of fatty acids using the basal ELFA as follows. The SID of EE and SID of AEE were determined using the same equation:

$$SID=\left[AID+\left(IFAend/FAf\right)\times 100\mathrm{\%}\right],$$

### Statistical analyses

All data were analyzed statistically using SAS 9.4 (SAS Inst. Inc., Cary, NC, USA). Homogeneity of variance was verified using the UNIVARIATE procedure of SAS. Dietary treatment was a fixed effect and pig and period were the random effects in the model. Individual pig was the experimental unit for all analyses. The LSMEANS procedure was used to calculate mean values of all dietary treatments. The *t*-test procedure was used to compare ileal and total tract basal endogenous losses. Data for AID and SID of fat and fatty acid were analyzed by ANOVA using the MIXED procedure of SAS. Multiple comparisons were conducted using Tukey’s test. The *t*-test procedure was used to compare the differences in calculated values for endogenous loss of EE and AEE, AID of EE and AEE or SID of EE and AEE. The relationship between SID of fat and fatty acid compositions in oils was determined using PROC CORR of SAS. Prediction equations for SID of fat in different oil sources were developed using PROC REG of SAS. Multivariate regression models were determined using stepwise selection with a significance stay level of 0.10. The adjusted coefficient of determination (*R*^2^_adj_), the Mallows statistic, root mean square error (RMSE), Akaike’s information criterion (AIC), and Bayesian information criterion (BIC) were used as the selection criterion for the best fit equations. The equations with the greatest *R*^2^_adj_ and the least RMSE, AIC and BIC were proposed to indicate the best fit. Differences were considered significant if *P* < 0.05.

## Results

Fecal and ileal digesta samples were obtained from most pigs during the collection period. However, one pig experienced loose stools during the third and fourth collection periods respectively, likely due to the higher fat content than that of their commonly-used diet and fecal and ileal digesta samples from these pigs were not included in the analysis.

### Fatty acid composition of different oil sources

The fatty acid composition of different oil sources varied greatly (Table 1). Coconut oil, the most saturated fat source used here, rich in C12:0 which accounts for 46% of total fatty acids. Palm oil was rich in saturated fatty acids (SFA, 46% of total fatty acids), particularly in C16:0 (40% of total fatty acids). The C18:1 was the main fatty acid for peanut oil, canola oil and rice oil (38%–46% of total fatty acids). The C18:2 was the main fatty acid for sunflower oil, corn oil, soybean oil and cottonseed oil (53%–61% of total fatty acids). The C18:3 was the main fatty acid for flaxseed oil (49% of total fatty acids).

### Basal endogenous losses of fat and fatty acids

The basal endogenous loss of acid-hydrolyzed fat at the end of the ileum and over the entire intestinal tract were not different (Table 3). The estimated basal endogenous losses of ether extract, C14:0, C15:0, C16:0, C17:0, C18:0, C20:0 and SFA over the entire intestinal tract were much greater (*P* < 0.05) than those at the end of the ileum, whereas C18:1, C18:2, C18:3 and unsaturated fatty acids (UFA) were just the opposite (*P* < 0.05). The endogenous loss of ether extract was lower than that of AEE (*P* < 0.01).

**Table 3:** Basal endogenous losses of fat and fatty acids at the end of the ileum and over the entire intestinal tract, g/kg DMI^1^

Basal endogenous losses	Ileum	Total tract	SEM	*P* value
EE^2^	3.14^B^	8.31^B^	0.564	< 0.001
AEE^2^	9.30^A^	11.26^A^	0.808	0.063
SFA				
C12:0	0.02	0.02	< 0.001	0.750
C14:0	0.02	0.17	0.031	< 0.001
C15:0	0.03	0.38	0.019	< 0.001
C16:0	0.70	1.08	0.104	0.011
C17:0	0.02	0.21	0.010	< 0.001
C18:0	0.22	0.90	0.110	< 0.001
C20:0	0.01	0.05	< 0.001	< 0.001
C21:0	0.01	0.01	0.006	0.419
C22:0	0.04	0.06	0.001	< 0.001
C23:0	0.02	0.03	< 0.001	0.010
C24:0	0.07	0.09	< 0.001	0.010
UFA				
C18:1	0.25	0.13	0.031	< 0.001
C18:2	0.41	0.12	0.037	< 0.001
C18:3	0.04	0.01	0.001	< 0.001
C20:1	0.02	0.02	0.010	0.102
C24:1	0.02	0.02	< 0.001	0.406
SFA	1.15	3.02	0.252	< 0.001
UFA	0.91	0.34	0.101	< 0.001

*DM* Dry matter intake, *EE* Ether extract, *AEE* Acid-hydrolyzed ether extract, *SFA* Saturated fatty acid, *UFA* Unsaturated fatty acid

^A,B^ Least squares means within a column with different superscripts differ (*P* < 0.01)

^1^ The estimated values for basal endogenous losses of fatty acids less than 0.01 g/kg DMI were not presented

^2^ Values for endogenous loss of EE and AEE were compared

### Ileal digestibility of fat and fatty acids

The C16:0, C18:0, C18:1 and C18:2 were the major components of fatty acids in dietary oil sources. Therefore, the differences in the digestibility of these four fatty acids across oil sources were compared. Differences in digestibility of other fatty acids were not compared, because the calculated digestibility of these fatty acids varied greatly when diets contained low concentrations of fatty acids. There was a significant difference in the AID of fat and fatty acids across the dietary oil sources except for C18:1 and C18:2 (Table 4; *P* < 0.05). The AID of AEE for canola oil was greater than that of palm oil (*P* < 0.05), and the fat digestibility of other 9 oils were not significantly different. The AID of SFA for coconut oil was greater than that of palm oil and soybean oil (*P* < 0.01). The average AID of fat across the 10 dietary fat sources was 84.66% (79.65% to 86.97%).

**Table 4:** Apparent ileal digestibility of fat and fatty acids in different oil sources, %

Item	EE	AEE	Fatty acid					
			C16:0	C18:0	C18:1	C18:2	SFA	UFA
Sunflower seed oil	94.03^a^	85.74^ab^	86.68^ab^	90.46^a^	96.92	98.38	85.93^ab^	98.45^a^
Peanut oil	93.86^a^	84.06^ab^	86.86^ab^	80.52^abc^	98.62	97.58	79.52^abc^	98.18^ab^
Corn oil	93.90^a^	85.76^ab^	89.92^a^	89.30^a^	97.16	98.12	85.50^ab^	97.96^abc^
Canola oil	95.54^a^	86.97^a^	86.18^ab^	89.71^a^	98.87	97.41	84.18^ab^	98.16^ab^
Rice oil	92.80^ab^	84.93^ab^	88.97^ab^	88.47^a^	97.35	97.84	85.43^ab^	97.03^abc^
Soybean oil	94.53^a^	84.87^ab^	83.26^abc^	67.94^bc^	97.57	94.95	77.89^bc^	96.63^abc^
Palm oil	89.80^b^	79.65^b^	70.98^c^	61.81^c^	95.44	94.64	69.78^c^	94.93^bc^
Cottonseed oil	93.43^ab^	84.32^ab^	75.68^bc^	73.12^abc^	93.65	95.82	80.22^abc^	94.62^c^
Flaxseed oil	93.70^a^	85.42^ab^	88.30^ab^	87.64^ab^	96.87	96.21	86.49^ab^	97.16^abc^
Coconut oil	92.96^ab^	84.83^ab^	78.49^abc^	75.95^abc^	93.27	94.48	91.10^a^	94.51^c^
SEM	0.728	1.282	2.690	3.972	2.407	0.834	2.539	0.662
*P* value	< 0.001	0.034	< 0.001	< 0.001	0.102	0.071	< 0.001	< 0.001

*EE* Ether extract, *AEE* Acid-hydrolyzed ether extract, *SFA* Saturated fatty acid, *UFA* Unsaturated fatty acids

^a,b,c^ Least squares means within a column with different superscripts differ (*P* < 0.05)

There was a significant difference in the SID of fat, C16:0, C18:0 and SFA across the dietary oil sources (Table 5; *P* < 0.01). The SID of AEE for palm oil was lower than that of sunflower oil, corn oil, canola oil, rice oil and flaxseed oil (*P* < 0.01). The AID of SFA for palm oil was lower than that of other oils (*P* < 0.01). The average in SID of fat across the 10 dietary fat sources was 96.5% (91.14% to 99.18%). Although the AID of EE was greater than that of AEE (*P* < 0.01), there was no significant difference in SID of EE and AEE except for palm oil (Table 6).

**Table 5:** Standardized ileal digestibility of fat and fatty acids in different oil sources, %^1^

Item	EE	AEE	Fatty acid					
			C16:0	C18:0	C18:1	C18:2	SFA	UFA
Sunflower seed oil	97.36^a^	96.87^a^	96.31^ab^	96.23^a^	98.62	99.58	96.86^a^	100.10
Peanut oil	97.26^a^	95.40^ab^	98.94^a^	93.33^a^	99.37	101.28	93.39^a^	99.97
Corn oil	97.49^a^	97.68^a^	97.48^a^	98.69^a^	98.93	99.49	97.85^a^	99.49
Canola oil	99.05^a^	99.18^a^	97.84^a^	98.01^a^	99.84	101.86	98.77^a^	99.80
Rice oil	97.11^a^	97.82^a^	97.66^a^	97.29^a^	99.56	101.50	96.70^a^	100.46
Soybean oil	97.57^a^	96.78^ab^	98.00^a^	93.12^a^	99.94	100.11	95.46^a^	100.36
Palm oil	93.25^b^	91.14^b^	76.00^c^	74.62^b^	97.54	102.71	76.79^b^	99.55
Cottonseed oil	96.97^a^	95.96^ab^	83.90^bc^	97.87^a^	99.25	98.28	92.20^a^	98.22
Flaxseed oil	97.81^a^	98.17^a^	97.41^ab^	93.26^a^	98.74	102.11	97.39^a^	100.60
Coconut oil	96.85^a^	95.75^ab^	92.26^ab^	92.75^a^	97.51	100.69	95.47^a^	100.61
SEM	0.708	1.162	2.941	3.130	0.873	0.655	2.649	0.633
*P* value	< 0.001	0.005	< 0.001	< 0.001	0.134	0.071	< 0.001	0.187

*EE* Ether extract, *AEE* Acid-hydrolyzed ether extract, *SFA* Saturated fatty acid, *UFA* Unsaturated fatty acids

^a,b,c^ Least squares means within a column with different superscripts differ (*P* < 0.05)

^1^ Standardized ileal digestibility of fat and fatty acids were calculated by correcting the apparent ileal digestibility values with the basal endogenous losses. Basal endogenous losses (g/kg DMI) averaged 3.14 EE, 9.30 AEE, 0.70 C16:0, 0.22 C18:0, 0.25 C18:1, 0.41 C18:2, 1.15 SFA, 0.91 UFA

**Table 6:** Effect of fat extraction methods on the apparent or standardized ileal digestibility of fat

Item	AID		SEM	*P* value	SID		SEM	*P* value
	EE	AEE			EE	AEE		
Sunflower seed oil	94.03	85.74	0.646	< 0.001	97.36	96.87	0.65	0.472
Peanut oil	93.86	84.06	0.742	< 0.001	96.46	95.40	0.74	0.120
Corn oil	93.90	85.76	1.402	< 0.001	97.49	97.68	1.43	0.807
Canola oil	95.54	86.97	1.610	< 0.001	99.05	99.18	0.44	0.719
Rice oil	92.80	84.93	1.367	< 0.001	97.11	97.82	0.45	0.157
Soybean oil	94.53	84.87	0.922	< 0.001	97.57	96.78	1.03	0.302
Palm oil	89.80	79.65	0.698	< 0.001	93.25	91.14	0.70	0.023
Cottonseed oil	93.43	84.32	0.478	< 0.001	96.97	95.96	0.43	0.110
Flaxseed oil	93.70	85.42	0.830	< 0.001	97.81	98.17	0.74	0.639
Coconut oil	92.96	84.83	1.444	< 0.001	96.85	95.75	1.15	0.367

*EE* Ether extract, *AEE* Acid-hydrolyzed ether extract, *AID* Apparent ileal digestibility, *SID* Standardized ileal digestibility

### Correlation analysis and prediction equations for SID of fat

Correlation coefficients between fatty acid composition and the SID of fat of the 10 oil sources were shown in Table 7. The ratio of unsaturated to saturated fatty acids (U/S) had a positive correlation with SID of fat (*P* < 0.05), whereas C16:0 and long chain saturated fatty acids (LSFA) were significant negatively correlated with SID of fat (*P* < 0.01). The stepwise regression equations and best prediction equation for SID of fat in different oil sources were presented in Table 8. The LSFA was the first predictor and C18:0 was the second predictor. Considering the accuracy of estimating the SID of fat, the equation that contained LSFA, C16:0 and C18:1 was more practical with the greatest *R*^2^_adj_ (0.88) and the lowest RMSE (0.75), AIC (−2.81) and BIC (−19.69). The best-fit equation to predict SID of fat was SID AEE = 102.75 − 0.15 × LSFA − 0.74 × C18:0 − 0.03 × C18:1 (*R*^2^_adj_ = 0.88, *P* < 0.01).

**Table 7:** Correlation coefficients between fatty acid composition and standardized ileal digestibility (SID) of fat in different oil sources

Item	C16:0	C18:0	C18:1n9c	C18:2n6c	MCFA	LSFA	LUFA	SFA	MUFA	PUFA	UFA	U/S
SID of EE	− 0.88^**^	− 0.50	− 0.14	0.26	− 0.04	− 0.86^**^	0.44	− 0.44	0.03	0.44	0.44	0.70^*^
SID of AEE	− 0.82^**^	− 0.60	− 0.09	0.27	− 0.12	− 0.86^**^	0.50	− 0.50	0.06	0.49	0.50	0.72^*^

*EE* Ether extract, *AEE* Acid-hydrolyzed ether extract, *MCFA* Medium-chain fatty acids (6−12 carbons), *LSFA* Long-chain saturated fatty acids (13−21 carbons), *LUFA* Long-chain unsaturated fatty acids (13−21 carbons), *SFA* Saturated fatty acids, *MUFA* Monounsaturated fatty acids, *PUFA* Polyunsaturated fatty acids, *UFA* Unsaturated fatty acids, *U/S* The ratio of unsaturated to saturated fatty acids

^*^ Means *P* < 0.05

^**^ Means *P* < 0.01

**Table 8:** Prediction equations of standardized ileal digestibility of fat in different oil sources^a^

Item	Equations^b^	Statistics^c^						
		*R*^2^	*R*^2^_adj_	C(p)	RMSE	AIC	BIC	*P* value
No.1	SID AEE = 99.58 − 0.15 × LSFA	0.73	0.70	123.09	1.21	5.63	2.24	0.002
No.2	SID AEE = 101.33 − 0.14 × LSFA − 0.74 × C18:0	0.85	0.80	70.41	0.98	2.13	− 2.57	0.001
No.3	SID AEE = 102.75 − 0.15 × LSFA − 0.74 × C18:0 − 0.03 × C18:1	0.92	0.88	37.76	0.78	− 2.14	− 7.25	< 0.001

^a^ The unit of fatty acid content in the equation was g/kg

^b^ *AEE* Acid-hydrolyzed ether extract, *SID* Standard terminal ileal digestibility, *LSFA* Long-chain saturated fatty acid (13 − 21 carbons)

^c^ *R*^*2*^_*adj*_ Adjusted coefficient of determination, *C(p)* the Mallows statistic, *RMSE* Root mean square error, *AIC* Akaike’s information criterion, BIC Bayesian information criterion

## Discussion

The objective of this experiment was mainly to determine the effect of fatty acid composition on the digestibility of dietary oil sources. In order to avoid other factors influencing the determined digestibility value, dietary oil sources used in the present study were all of good quality. The fatty acid composition of oils used in the present experiment were similar to those shown by NRC [13].

Basal ELF and ELFA at the end of the ileum and over the entire intestinal tract were similar to our previous results [11]. Even when experimental conditions, such as fat-free diet composition, sample collection, and analytical procedures, were strictly controlled, small differences in basal ELF and ELFA can be observed between the two experiments. Therefore, we recommended that basal ELF and ELFA can be estimated routinely in studies aimed at evaluating SID of fat and fatty acids. In addition, the basal endogenous losses over the entire intestinal tract were also measured in the present experiment to verify the accuracy of the results of the fat-free diet method. Because it is more accurate to measure digestibility values of fat and fatty acids at the end of the ileum [11], the endogenous losses over the entire intestinal tract need not be determined in future studies.

The ELFA differences between the terminal ileum and the entire intestinal tract were consistent with previous results [11, 20, 21]. In the present experiment, the amount of endogenous fat loss should be constant. However, due to differences in fat extraction methods, endogenous loss of acid-hydrolyzed fat at the end of the ileum was not different from that over the entire intestinal tract, while the endogenous loss of ether extract was greater for the entire intestinal tract than at the end of the ileum. The reason for this observation may be that the contents of combined fat in the ileal samples were greater than those in the fecal. Samples were analyzed using a solvent extraction procedure without acid hydrolysis cannot completely extract fat and fatty acids, especially if they were linked to proteins or carbohydrates, or were present as salts of divalent cations (such as Ca-fatty acid soap) [22]. Fat and fatty acids that cannot be completely extracted by crude fat extraction method are defined as combined fat. Compared to the acid hydrolysis process, direct extraction was believed to result in incomplete extraction of combined fat [23]. Therefore, the underestimated fat contents were greater in the ileal samples than in the fecal samples, resulting in less endogenous loss of ether extract in the terminal ileum than for the entire intestinal tract. This result was consistent with that reported by Chen et al. [21]. In addition, some studies reported that the endogenous loss of acid-hydrolyzed fat over the entire intestinal tract were greater than that at the end of the ileum [14, 24]. However, no difference in endogenous loss of acid-hydrolyzed fat was observed between the two collection sites, most likely because the beet pulp used in the present experiment was rich in soluble dietary fiber [11]. Soluble dietary fiber may increase the synthesis of endogenous fat by microbes in the ileum of pigs.

The AID of fat was greater for canola oil compared with palm oil. This was due to the greater content of UFA for canola oil whereas the greater content of SFA for palm oil. Unsaturated fatty acids are considered less hydrophobic and more soluble when exposed to bile salts compared with SFA [25]. This chemical property may increase incorporation of UFA into the micelles with greater efficiency than SFA and facilitate their subsequent absorption [1, 25]. The average value for SID of UFA (99.92%) was greater than that of SFA (94.09%), which further confirms that UFA is more digestible than SFA. Although coconut oil had the highest content of SFA (89.90%, of total fatty acids), the SID of fat for coconut oil was close to that of cottonseed oil. This is due to the fact that coconut oil is rich in medium chain fatty acids (MCFA), which are hydrolyzed faster than LCFA [26, 27]. Medium chain fatty acids can theoretically be passively absorbed through the gastrointestinal mucosa without the formation of mixed micellar [28]. Therefore, the digestibility of MCFA was greater than that of LCFA [29]. Previous studies had also shown that the digestibility of fat was relatively high when pigs fed diet containing coconut oil compared to diet containing corn oil [30] or palm oil [31] in piglets. Except for palm oil, there were no differences in AID of fat among the other 9 oils in the present experiment. This result agrees with the observation from Jørgensen et al. [32]. Previous study reported that the AID of C18:0 in pigs fed diets containing fish oil, canola oil and coconut oil was 36.3%, 11.3% and −16.9%, respectively [32]. However, in the present experiment, the AID of C18:0 in different oil sources was more than 60%. This difference may be a result of different content and characteristic of fat in basal diet (Intact fat; 3.90% versus 0.29%) in the previous experiment compared with the present experiment. Intact fat is encased with cell membranes or fibrous compounds and thus it is more resistant to enzymatic digestion than extracted fat [14, 33, 34]. Therefore, the ATTD of C18:0 in extracted oil were greater than in their corresponding intact oil [35].

It is important to specify the procedures used for fat analyses [36]. However, no studies have compared the differences in calculated values for endogenous loss and digestibility of EE and AEE. The results of this experiment indicated that greater ELF and lower AID of fat were observed using the acid hydrolysis procedure compared to the direct solvent extraction procedure. The acid hydrolysis procedure was thought to result in a more complete extraction of fat [22]. Therefore, the solvent extraction method without acid hydrolysis can result in lower analyzed values for fat in all samples. In addition, because the contents of combined fat in the ileal or fecal samples were greater than those in diets, the underestimated fat contents in the ileal or fecal samples were greater than in diet samples [14]. These differences explained why the endogenous loss of EE was less than that of AEE and the AID of EE was greater than that of AEE in the present experiment. However, the fat extraction method had no effect on the SID of fat except for palm oil. Collectively, when calculating the SID of fat, the EE content of the samples can be analyzed using the direct extraction method, whereas the acid hydrolysis procedure should be used to determine the AID of fat.

There were fewer reports on the SID of fat and fatty acid for different oil sources due to the limitation of basal ELF and ELFA measurement. The SID of fat and fatty acids for soybean oil in the present experiment were slightly greater than the SID values reported by Wang et al. [11]. Variation in SID of fat across experiments may be due to differences in body weight, dietary fat intake, diet processing and diet composition. The results of this experiment showed that the SID of fat across all oil sources were more than 90%, which indicates that the dietary fat is an extremely digestible energy feed with high absorption efficiency in the digestive tract of animals [1, 32, 37]. Previous research regarded the fat-free diet method as the proper approach to determining the standardized digestibility of fat and fatty acids in plant oils [11, 38]. The present experiment was the first one to comprehensively determine the SID of fat and fatty acids from ten oil sources using the fat-free diet method. These findings are of great significance for the determination of fatty acid requirements and effective utilization of oil in commercial practice. In addition, the method for determining the SID of fat and fatty acids was clarified. Therefore, it is suggested that basal ileal endogenous losses of fat and fatty acids should be measured in digestibility experiments and SID values should be calculated.

The C16:0 and LSFA were negatively correlated with SID of fat. As previously explained, fatty acids with shorter chain lengths and (or) higher degree of unsaturation are digested and absorbed faster and more completely [39, 40], leading to greater digestibility coefficients. In addition, LSFA are more easily to complex with free Ca ions and form Ca-fatty acid soaps compared with unsaturated fatty acids within the gastrointestinal tract [41, 42]. The formation of highly insoluble Ca-fatty acid soap results in decreased fat absorption in the gastrointestinal tract of growing pigs [43]. Although U/S was positively correlated with SID of fat, U/S was not a good predictor. This may be because coconut oil had the lowest U/S, but its digestibility was not the lowest, even greater than palm oil and peanut oil.

Developing prediction equations for SID of fat is essential to rapidly and precisely determine the digestibility of dietary oil sources. Previous studies have successfully established prediction equations for energy values of dietary oil sources in pigs [8–10]. The present study was the first one to establish prediction equations for SID of fat based on the measured fatty acid composition in dietary oil sources fed to growing pigs. The content of LSFA and C18:0 of oils was the first and second predictors, which is closely associated with correlation analysis. The best-fit equation that included LSFA, C18:0 and C18:1 showed the greatest *R*^2^_adj_ and the lowest RMSE, AIC and BIC, which indicated that the fatty acid composition could be used to predict the SID of fat in dietary oil sources fed to growing pigs. Notably, the prediction equations are likely accurate for the oil samples from which they were generated, but may not be accurate for estimating the SID of fat when applied to additional oil samples. Therefore, more SID data of oil sources should be determined in future studies to verify the precise and accuracy of the prediction equations.

## Conclusions

When calculating the SID of fat, the EE content of the samples can be analyzed using the direct extraction method, whereas the acid hydrolysis procedure should be used to determine the AID of fat. There were differences in the fatty acid composition as well as the AID and SID of fat and SFA across the 10 oil sources. Fat digestibility of dietary oils was related to their fatty acid composition and especially to their contents of C16:0, LSFA and U/S. The best-fit equation to predict SID of fat was SID AEE = 102.75 − 0.15 × LSFA − 0.74 × C18:0 − 0.03 × C18:1 (*R*^2^_adj_ = 0.88, *P* < 0.01).

## Supplementary Information

**Additional file 1:** **Table S1.** Ingredient composition (%, as-fed basis) ofexperimental diets.

## Data Availability

The data analyzed during the current research are available from the corresponding author on reasonable request.

## Abbreviations

ADF: Acid detergent fiber
AEE: Acid-hydrolyzed fat
AIC: Akaike’s information criterion
AID: Apparent ileal digestibility
BIC: Bayesian information criterion
CP: Crude protein
DM: Dry matter
EE: Ether extract
ELF: Endogenous losses of fat
ELFA: Endogenous losses of fatty acids
LSFA: Long chain saturated fatty acids
MCFA: Medium chain fatty acids
NDF: Neutral detergent fiber
RMSE: Root mean square error
SFA: Saturated fatty acids
SID: Standardized ileal digestibility
U/S: The ratio of unsaturated to saturated fatty acids
UFA: Unsaturated fatty acids
