# Supplementary material for: Effects of dietary oil sources and fat extraction methods on apparent and standardized ileal digestibility of fat and fatty acids in growing pigs
Source: J Anim Sci Biotechnol. 2022 Dec 14;13:143. doi: 10.1186/s40104-022-00798-w (PMC9749213; doi:10.1186/s40104-022-00798-w)
Supplement: Supplementary file 1 — Additional file 1: Table S1. Ingredient composition (%, as-fed basis) ofexperimental diets. [file 40104_2022_798_MOESM1_ESM.docx]

**Table S1 Ingredient composition (%, as-fed basis) of experimental diets**

| Item | Fat-free diet | Oil-added diets | | | | | | | | | |
| --- | --- | --- | --- | --- | --- | --- | --- | --- | --- | --- | --- |
|  |  | Sunflower seed oil | Peanut oil | Corn oil | Canola oil | Rice oil | Soybean oil | Palm oil | Cottonseed oil | Flaxseed oil | Coconut oil |
| Cornstarch | 44.00 | 44.00 | 38.00 | 38.00 | 38.00 | 38.00 | 38.00 | 38.00 | 38.00 | 38.00 | 38.00 |
| Soy protein isolate | 16.00 | 16.00 | 16.00 | 16.00 | 16.00 | 16.00 | 16.00 | 16.00 | 16.00 | 16.00 | 16.00 |
| Oil source^1^ | 0.00 | 6.00 | 6.00 | 6.00 | 6.00 | 6.00 | 6.00 | 6.00 | 6.00 | 6.00 | 6.00 |
| Sucrose | 10.00 | 10.00 | 10.00 | 10.00 | 10.00 | 10.00 | 10.00 | 10.00 | 10.00 | 10.00 | 10.00 |
| Sugar beet pulp^2^ | 26.00 | 26.00 | 26.00 | 26.00 | 26.00 | 26.00 | 26.00 | 26.00 | 26.00 | 26.00 | 26.00 |
| Dicalcium phosphate | 2.00 | 2.00 | 2.00 | 2.00 | 2.00 | 2.00 | 2.00 | 2.00 | 2.00 | 2.00 | 2.00 |
| Limestone | 0.20 | 0.20 | 0.20 | 0.20 | 0.20 | 0.20 | 0.20 | 0.20 | 0.20 | 0.20 | 0.20 |
| Sodium chloride | 0.40 | 0.40 | 0.40 | 0.40 | 0.40 | 0.40 | 0.40 | 0.40 | 0.40 | 0.40 | 0.40 |
| Chromic oxide | 0.40 | 0.40 | 0.40 | 0.40 | 0.40 | 0.40 | 0.40 | 0.40 | 0.40 | 0.40 | 0.40 |
| Vitamin-mineral premix^3^ | 0.50 | 0.50 | 0.50 | 0.50 | 0.50 | 0.50 | 0.50 | 0.50 | 0.50 | 0.50 | 0.50 |
| *L*-Lys HCl (78.8%) | 0.20 | 0.20 | 0.20 | 0.20 | 0.20 | 0.20 | 0.20 | 0.20 | 0.20 | 0.20 | 0.20 |
| *DL*-Met (98.5%) | 0.12 | 0.12 | 0.12 | 0.12 | 0.12 | 0.12 | 0.12 | 0.12 | 0.12 | 0.12 | 0.12 |
| *L*-Thr (98.5%) | 0.18 | 0.18 | 0.18 | 0.18 | 0.18 | 0.18 | 0.18 | 0.18 | 0.18 | 0.18 | 0.18 |
| Total | 100.00 | 100.00 | 100.00 | 100.00 | 100.00 | 100.00 | 100.00 | 100.00 | 100.00 | 100.00 | 100.00 |

^1^ Oils were provided by Zhongda Agricultural Science and Technology Co., Ltd. (Jinan, China).

^2^ Sugar beet pulp was provided by Hefeng Feed Co., Ltd. (Beijing, China).

^3^ Vitamin-mineral premix provided the following per kg of complete diet for growing pigs: vitamin A, 5512 IU; vitamin D_3_, 2200 IU; vitamin E, 30 IU; vitamin K_3_, 2.2 mg; vitamin B_12_, 27.6 μg; riboﬂavin, 4.0 mg; pantothenic acid, 14.0 mg; niacin, 30.0 mg; choline chloride, 400.0 mg; folacin, 0.7 mg; thiamine 1.5 mg; pyridoxine 3.0 mg; biotin, 44.0 μg; Mn, 40.0 mg (MnO); Fe, 75.0 mg (FeSO_4_·H_2_O); Zn, 50.0 mg (ZnO); Cu, 15.0 mg (CuSO_4_·5H_2_O); I, 0.3 mg (KI); Se, 0.3 mg (Na_2_SeO_3_)
